# Recent Advances in Carbon Nanotube Utilization in Perovskite Solar Cells: A Review

**DOI:** 10.3390/mi15040529

**Published:** 2024-04-15

**Authors:** Usman Asghar, Muhammad Azam Qamar, Othman Hakami, Syed Kashif Ali, Mohd Imran, Ahmad Farhan, Humaira Parveen, Mukul Sharma

**Affiliations:** 1 Center of Excellence in Solid State Physics, University of the Punjab, Lahore 54590, Pakistan; usmanasghar25071988@gmail.com; 2 Department of Chemistry, School of Science, University of Management and Technology, Lahore 54770, Pakistan; 3 Department of Physical Sciences, Chemistry Division, College of Science, Jazan University, P.O. Box 114, Jazan 45142, Saudi Arabia; omhakami@jazanu.edu.sa; 4 Nanotechnology Research Unit, College of Science, Jazan University, P.O. Box 114, Jazan 45142, Saudi Arabia; 5 Department of Chemical Engineering, College of Engineering, Jazan University, P.O. Box 706, Jazan 45142, Saudi Arabia; malain@jazanu.edu.sa; 6 Department of Chemistry, University of Agriculture Faisalabad, Faisalabad 38000, Pakistan; gghssvehari@gmail.com; 7 Department of Chemistry, Faculty of Science, University of Tabuk, Tabuk 71491, Saudi Arabia; h.nabi@ut.edu.sa; 8 Environment and Nature Research Centre, Jazan University, P.O. Box 114, Jazan 45142, Saudi Arabia; mukulsh77@gmail.com

**Keywords:** perovskite solar cells, carbon nanotubes, conducting substrate, green energy

## Abstract

Due to their exceptional optoelectronic properties, halide perovskites have emerged as prominent materials for the light-absorbing layer in various optoelectronic devices. However, to increase device performance for wider adoption, it is essential to find innovative solutions. One promising solution is incorporating carbon nanotubes (CNTs), which have shown remarkable versatility and efficacy. In these devices, CNTs serve multiple functions, including providing conducting substrates and electrodes and improving charge extraction and transport. The next iteration of photovoltaic devices, metal halide perovskite solar cells (PSCs), holds immense promise. Despite significant progress, achieving optimal efficiency, stability, and affordability simultaneously remains a challenge, and overcoming these obstacles requires the development of novel materials known as CNTs, which, owing to their remarkable electrical, optical, and mechanical properties, have garnered considerable attention as potential materials for highly efficient PSCs. Incorporating CNTs into perovskite solar cells offers versatility, enabling improvements in device performance and longevity while catering to diverse applications. This article provides an in-depth exploration of recent advancements in carbon nanotube technology and its integration into perovskite solar cells, serving as transparent conductive electrodes, charge transporters, interlayers, hole-transporting materials, and back electrodes. Additionally, we highlighted key challenges and offered insights for future enhancements in perovskite solar cells leveraging CNTs.

## 1. Introduction

The use of renewable and sustainable energy sources is gaining popularity [1]. Rising concerns about climate change and the depletion of fossil fuel reserves are the primary motivators here [2,3,4,5]. Solar power is an affordable and practical way to meet the energy demands of the future without sacrificing viability [6,7]. The optoelectronic potential of metal halide perovskites is rapidly expanding [8,9]. Their remarkable optoelectronic properties, including a variable bandgap, a large absorption coefficient, and a high carrier mobility, are largely responsible for this. Researchers have also shown that they have a long-balanced carrier diffusion length and very little nonradiative loss [10]. Numerous optoelectronic devices, such as photodetectors, light-emitting diodes, and solar cells, benefit greatly from their unique properties [11,12]. Emerging as a very promising alternative to the prevailing silicon-based photovoltaic technology, metal halide perovskite solar cells are a new breed of photovoltaic devices. The reason for this is that research can be performed using cost-effective, scalable, low-temperature, solution-processing methods [13,14].

Either a p-i-n or n-i-p configuration of a single-junction perovskite solar cell will typically have the following components: a back electrode, a perovskite light absorber layer, an ETL, and a transparent conductive metal oxide electrode. An often-used substrate for subsequent layer deposition is a transparent conductive metal oxide that covers a rigid plastic or glass support. Whether it is by solution-based or thermal evaporation, the perovskite layer is sandwiched between the two electron transport layers (ETLs) and the hole transport layer (HTL). To finish the perovskite solar cell, the metal back electrode is vaporized. To achieve the best possible performance from their devices, researchers have repeatedly tweaked the perovskite composition and crystal growth, improved the device interfaces, and created new device topologies [15,16].

In a relatively short span of time, the global scientific community has made significant efforts to enhance the photovoltaic conversion efficiency of perovskite solar cells, achieving a notable rise from 3.8% to 30%. The crystalline silicon solar cell continues to exhibit a good commercial performance due to its limited stability and inadequate scalability. The majority of perovskite solar cells (PSCs) with a high efficiency documented in the literature are based on an area of 0.01 cm^2^. It has been shown that the efficiency of PSCs diminishes as the area increases [17,18,19]. The perovskite/silicon heterojunction tandem solar cell efficiency has reached 28.6% [20], and the perovskite/perovskite tandem solar cell efficiency has crossed 32% [21], while the theoretical maximum efficacy reported is over 35% [22].

Perovskites made of metal halides dissolve readily in a wide range of polar solvents due to their strong ionic bonding and low formation enthalpy. Because of this quality, study samples may be effectively and affordably fabricated at low temperatures for use in solution processing. Because of their remarkable fault tolerance, films made via solution processing retain the unique optoelectronic properties of perovskites. Because of this, they cannot be processed like regular semiconductors, which need a high-temperature vacuum [23].

Spray deposition, ink-jet printing, gravure printing, blade coating, and slot-die coating are some of the scalable manufacturing technologies that have been used to produce large-area PSCs [24,25]. With thermally co-evaporated deposition, a minimodule of 21 cm^2^ was able to obtain a power conversion efficiency of over 18% [26]. In addition, semitransparent perovskite solar cells may be readily created using perovskites due to their high absorption coefficient and bandgap adjustment capabilities. Building and vehicle windows and facades that generate electricity might be a promising use for these PSCs [27,28]. Aside from the continuous endeavors to attain opaque and semitransparent PSCs with high-performance single junctions, there is an increasing emphasis on creating tandem solar cells based on perovskites. With these cells, researchers hope to significantly enhance the cost-efficiency ratio of solar technology while simultaneously reducing the spectral losses [29,30].

The power conversion efficiencies of perovskite–silicon tandem solar cells are now 28% under one sun, whereas perovskite–perovskite solar cells are 24.8% efficient [31]. Progress has been made in the device designs and power conversion efficiencies, but there is still a long way to go before perovskite solar cells reach their full potential. These cells need to be more resistant to degradation, cheaper, and lead-free [32]. The importance of addressing the stability issue in PSCs has been extensively recognized [33,34]. Their stability might be affected by environmental factors such as ultraviolet light, thermal stress, and moisture penetration. Metal electrodes and selective charge transport layers are two internal factors that greatly impact the device’s long-term functioning. In recent years, there have been notable improvements in the stability of the devices [35,36].

Many variables affect the energy payback time of PSCs, including stability and the price of materials and manufacturing. Presently, one of the most expensive components of PSCs is transparent conductive oxide electrodes, which are typically composed of indium tin oxide or fluorine tin oxide [37]. Researchers agree that they are not ideal for use in flexible PSCs because of their low mechanical flexibility. Furthermore, the application of metal back electrodes, usually composed of silver (Ag) or gold (Au), may be an expensive and energy-intensive process that requires high-vacuum evaporation processes. On top of that, when the device is operating, undesirable electron and hole recombination often occurs inside the perovskite layers and at their interfaces, limiting the enhancement of device performance [38]. Using new materials and improving the designs of the existing components are key to solving these issues.

A lot of carbon materials have been employed to fix the problems with PSCs that were mentioned before [39]. Particularly intriguing to researchers working on PSCs are carbon nanotubes (CNTs), a kind of one-dimensional carbon nanomaterial. Carbon nanotubes have found several applications in various electrical and optoelectronic devices since the early 1990s [40,41]. The structural view of carbon nanotubes suggests they are essentially hollow cylinders made by winding up graphene sheets in one or more layers. Carbon nanotubes may be categorized into three main types: single-walled, double-walled, and multi-walled nanotubes [42]. By looking at their chiral angles (θ), SWCNTs may be grouped into three types: zigzag (θ = 0°), chiral (0° < θ < 30°), and armchair (Ÿ = 30°) [43]. In addition, the diameters and chiral angles of SWCNTs define whether they are metallic or semiconducting. The electrical conductivity of metallic single-walled carbon nanotubes is usually between 104 and 106 S m^−1^. At room temperature, their thermal conductivity may go as high as 3500 W m^−1^ K^−1^ [44].

Carbon nanotubes have a tensile strength about 100 times higher than steel and an extraordinarily large Young’s modulus ranging from 1 to 4 TPa, both of which are caused by the existence of covalent sp^2^ bonds between the carbon atoms [45]. The key properties of carbon nanotubes have been briefly described in a number of articles. The remarkable use of carbon nanotubes in various perovskite solar cells is due to their enthralling optical and electrical properties and extraordinary mechanical flexibility. Among them, you may find PSCs that have a mesoporous n-i-p structure, a planar n-i-p structure, an inverted p-i-n structure, or a mesoscopic structure that is devoid of a hole transport layer. There has recently been a lot of success in using carbon nanotubes for different parts of devices to make perovskite solar cells work better. The constituent parts of perovskite light absorbers include additives, interlayers, transparent conductive electrodes, back electrodes, materials for transporting electrons and holes, and interlayers [46,47].

The most impressive power conversion efficiency of more than 25% was achieved by completely incorporating carbon nanotubes into perovskite solar cells (PSCs). As a consequence of these encouraging findings, we are re-evaluating the role of carbon nanotubes in PSCs. There have been some encouraging reviews of carbon materials used in PSCs in recent papers, but no comprehensive research has yet addressed CNTs in PSCs. So, in this review, we summarize and critically evaluate the most recent developments in incorporating carbon nanotubes into perovskite solar cells. Our goal here is to provide readers with a synopsis of the recent progress in carbon nanotube (CNT)-based PSCs. Finally, we highlight the key challenges and provide our thoughts on how perovskite solar cells based on carbon nanotubes may be improved in the future [48].

## 2. Carbon Nanotube Structure

Fabricated artificially, carbon nanotubes are an allotrope of carbon. The two-dimensional graphene may be considered as a parent material for fullerenes, carbon nanotubes, and three-dimensional graphite [49]. The high area ratio and hollow cylindrical form of carbon nanotubes are its defining features. The other atoms making up the tube’s walls are sp^2^ hybrids and others are sp^3^ hybrids, creating a state of mixed hybridization. There are four atoms linked to the sp^3^ hybrid orbital (bent at a 109.5° angle) and three atoms linked to the sp^2^ hybrid orbital (bent at a 120° angle), respectively. Linear defects with sp^3^ hybrid orbitals are present in the sp^2^ hybrid lattice. A carbon nanotube environment maintains this structure. If a carbon nanotube surface has five-membered rings, the form is convex; if the surface has seven-membered rings, the shape is concave. Because of this, the carbon nanotube’s body becomes uneven and flattened. A carbon nanotube’s capped end is created when the tube’s top is adorned with a heptacyclic or pentacyclic ring. It is common for seven or five ring members to enclose the cylinder in big fullerene and hemispherical molecules [50].

The number of wall layers allows for carbon nanotubes to be classified as either single- or multi-walled carbon nanotubes. When compared to SWCNTs, MWCNTs are more chemically inert. On top of that, multi-walled carbon nanotubes have cleaner surfaces. Nanotube radii are inversely proportional to shear and Young’s moduli [51]. Defect centers, which resemble rings with seven or five members, commonly develop at the extremities of the tubes, on the surfaces of the layers, and between the layers of single-walled carbon nanotubes as they expand. The chemical reactions caused by flaws get more intense, and the surface’s chemical composition becomes more complex as the number of carbon nanotube layers increases [52]. Multi-walled carbon nanotubes, which make up the vast majority of CNTs, are composed of several coaxial cylindrical tubes with several layers ranging from multiples of layers to tens of layers. In addition, other academic articles have shed light on various carbon nanotube structures and applications.

**Figure 1:**
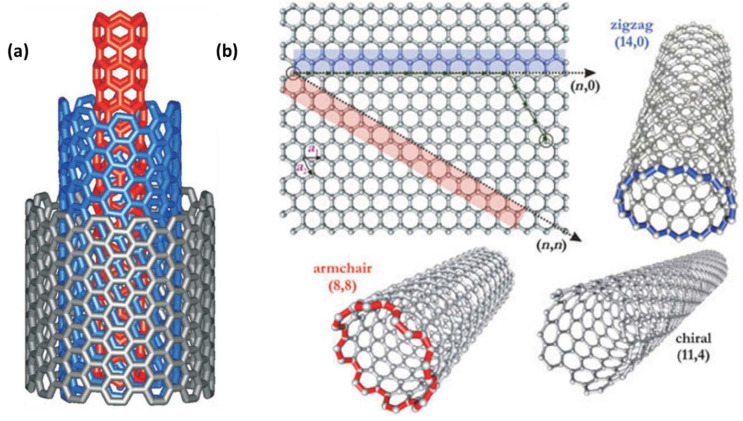
(**a**) A multi-walled carbon nanotube structure composed of three shells of varying chirality. (**b**) A graphene sheet is rolled up, resulting in three distinct forms of CNTs. Reproduced with permission from [56]. Copyright (2004) John Wiley and Sons.

The chiral vector, abbreviated as Ch, is a common way to describe the atomic arrangement on single-walled carbon nanotubes. Where n and m are the chiral indices, the chiral vector is given by Ch = na^1^ + ma^2^. Armchair, chiral vectors, and zigzag formations are shown by the big arrows. It is possible to divide SWCNTs into three distinct types according to their axial orientations (n, m): zigzag form (m = 0 or n = 0), armchair form (n = m), and chiral form (n ⋦ m). Carbon nanotubes come in three different forms, as seen in the picture [53]. Chairs may have many structures, such as armchair, zigzag, chiral, double-walled, and multi-walled types (Figure 1) [54,55].

## 3. Carbon Nanotube Synthesis Methods

### 3.1. Laser Ablation

The use of a laser to create nanotubes is another method. Producing nanotubes involves, as the name suggests, the use of a laser. A more advanced technology that overcomes the electric arc method’s limitations is the laser ablation approach. This is relevant to the production of both MWCNTs and SWCNTs, or single-walled carbon nanotubes. Nanotubes are often mass produced using this method. Their synthesis involves abrading the carbon target using a pulsed laser. A catalyst and an inert gas are used to execute pulsed laser ablation on a graphite target. The procedure culminates in the formation of carbon nanotubes. Ropes of SWCNTs were created by Thess et al. using the laser ablation process [57].

In these experiments, nanotubes with lengths of hundreds to several microns and sizes of 5 to 20 nm were created. According to researchers, carbon nanotube structures may be verified using transmission electron microscopy and X-ray diffraction methods. Direct ablation and carbon particle suspension in the reaction zone are the two suggested ways to fabricate carbon nanotubes [58]. Additionally, it has been shown that the synthesis of SWCNTs is negatively affected by the employment of a high-intensity UV laser. Several factors impact the laser ablation-based synthesis, including the catalyst, laser power, temperature, wavelength, inert gas selection, fluid dynamics, and pressure around the carbon target. Maintaining control over each of these variables is critical during nanotube synthesis. The production of carbon nanotubes using the laser ablation method was shown to have many flaws. The high final temperature of the synthesized molecules is one disadvantage.

**Figure 2:**
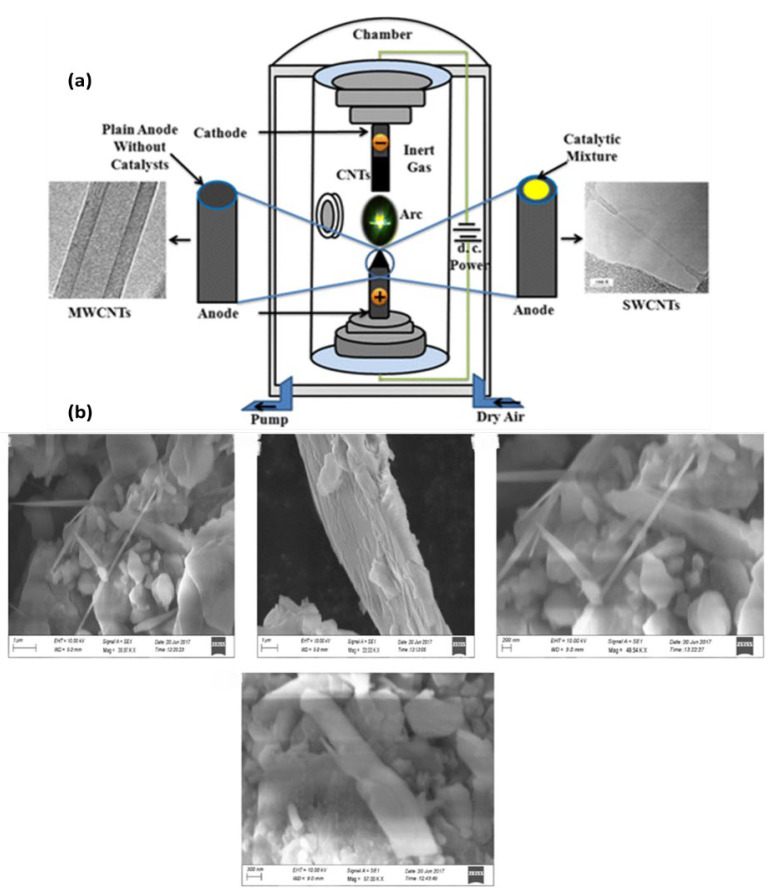
(**a**) Schematic of AD apparatus for synthesizing SWCNTs and MWCNTs. (**b**) SEM images of carbon nanotube. Reproduced with permission from [61]. Copyright (2016) Springer Nature.

Faizah A. AlMalki et al. used a Q-switched pulsed Nd laser. A graphite target immersed in deionized water (D-W) was ablated using a YAG laser operating at a wavelength of 1064 nm. The laser had a pulse repetition rate of 1 Hz and a pulse duration of 9 ns (mm). A graphite pellet with a purity of 99.91% (obtained from National Spectroscopic Electrodes Co.) and a diameter of mm was placed at the bottom of a glass jar filled with 3 mL of deionized water (D-W). The laser energy varied between 60 and 220 mJ, with 75 pulses [59,60]. The production of pristine single-walled carbon nanotubes (SWCNTs) is much sought after due to their extensive use in the sectors of electronics and biomedicine. To synthesize this particular tube using the AD approach, the anode was immersed in different metal catalysts that are unnecessary for the creation of MWCNTs (Figure 2a) [61].

### 3.2. Hydrothermal/Sonochemical Method 

Using a sonochemical technique, Jeong et al. produced SWCNTs. The catalyst, ferrocene, was combined with the carbon source, p-xylene, and the nucleation site, silica powder, to collect CNTs following ultrasonication at room temperature [62]. The sonochemical approach was used by Raja and Ryu to synthesize CNTs. Sonicating dichlorobenzene and ZnCl_2_ particles in an ultrasonic water bath was a straightforward process. The system’s maximum output was 50 W at 40 KHz [63]. Sodium hydroxide, distilled water, polyethylene glycol, and ethyl alcohol were among the precursor materials used in the hydrothermal synthesis of CNTs by Wang et al. In a 250 mL flask, these ingredients were coupled. Using a magnetic stirrer, the reaction mixtures were vigorously mixed for 30 min. Once the first half hour was over, the precursor was transferred to a reactor to undergo further treatment for 20 h at 160 °C. The researchers removed the material when it cooled [64]. The hydrothermal approach was used by Razali et al. to synthesize CNTs. The carbon sources, catalyst, and solvent utilized were ferrocene (2 g), sulfur (4 g), and a NaOH solution (10 MW). For 24 h, the precursor was heated to 200 °C in an autoclave reactor made of Teflon–stainless steel. The samples were collected for further analysis after chilling [65]. Carbon nanotubes (CNTs) are a recent development in nanotechnology has and have become a cornerstone of scientific study owing to their extensive use in the fields of spintronics, optical materials, and optoelectronics. Currently, carbon nanotubes (CNTs) are extensively produced using a variety of well-established techniques that include elevated temperatures. Carbon nanotubes (CNTs) were produced utilizing a hydrothermal approach at a low temperature of 160 °C. The precursors used for this synthesis were ethanol, polyethylene glycol (PEG), and sodium hydroxide. The reaction time for the synthesis was 20 h. The structure of the prepared CNTs was evaluated using a SEM analysis (Figure 2b) [66].

### 3.3. Arc Discharge

Nanotube production dates back to the earliest known technology, the arc discharge process. The most effective method was believed to be arc evaporation for synthesizing multi-walled carbon nanotubes [67]. To create an electric arc, the arc discharge process calls for two electrodes and a direct current to be sent between them [68]. The process begins by placing the electrodes within a chamber that is vacuumed and then filled with inert gas. Carbon may be quickly deposited on the electrodes due to the constant gas flow. Having plasma within the chamber helps with this deposition process. Two critical factors are (1) the pressure of the inert gas and (2) the current which must be regulated precisely for the process to work. While the power source is activated, the inert gas pressure within the reaction chamber is kept constant. After that, an electric arc is created when the positive and negative electrodes get close to each other, crash, and then separate. The electrodes produce plasma when they are heated to a high degree via arcing. Afterward, carbon nanotubes begin to cling to the negative electrodes. Using this approach, carbon nanotubes with one or more walls may be produced, as can carbon nanotubes with multiple walls. In contrast to MWCNTs, which are produced simply by controlling the pressure of the inert gas, SWCNTs need a catalyst to be used in their production. Using this method, nanotubes with a length of several microns and a diameter of 2–20 nm may be grown [69].

### 3.4. Electrolysis Method

**Figure 3:**
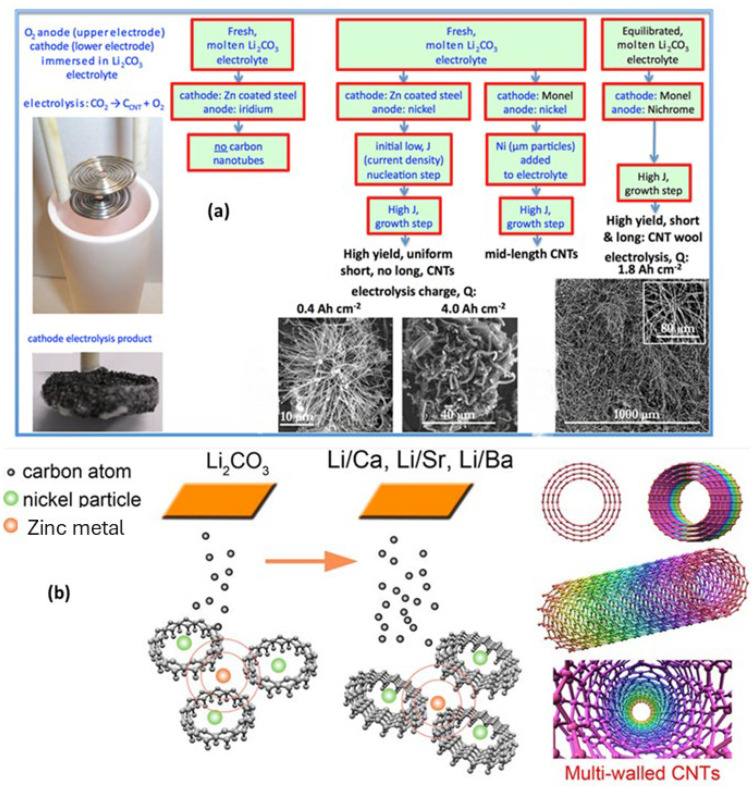
(**a**) Schematic depiction of novel synergistic routes for the electrolysis of CNT “wool” of a macroscopic length in molten carbonate, leading to a high yield. Reproduced with permission from [71]. Copyright (2017) Elsevier. (**b**) Methods for producing CNTs in solutions containing either pure Li_2_CO_3_ or salts including alkaline earth carbonate. Reproduced with permission from [72]. Copyright (2019) IOP Publishing.

Carbon nanotubes were created by researchers using electrochemical deposition. Utilizing a tri-chamber electrochemical cell, an electrochemical reactor was built. Nanoparticles coated with an Fe/Ni alloy were employed to form the anode and cathode, with the calomel electrode serving as the standard reference. In terms of its volume, the electrode was composed of a mixture of 40% methanol and 60% benzyl alcohol. The cathode was responsible for producing carbon nanotubes due to the application of a potential difference of about 1000 volts between the anode and cathode [70]. Carbon nanotubes were synthesized by Johnson and colleagues using molten electrolysis, with CO_2_ as the initial material (Figure 3a). For synthesis purposes, molten lithium carbonate was used. A crucible constructed of pure alumina was used to carry out the reaction. A variety of metals served as the cathode, while nickel served as the anode. The cathode formed carbon nanotubes, whereas the anode formed oxygen as a consequence of electrolysis. Two steps, including dissolution and electrolysis, were used to carry out the synthesis strategy. In the course of dissolution, lithium carbonate was generated. Afterward, CNTs were formed from lithium carbonate [71]. Through the use of CO_2_-based molten salt electrolysis, Li et al. were able to synthesize carbon nanotubes. In this specific method, Li_2_CO_3_, CaCO_3_, SrCO_3_, and BaCO_3_ were used in the CNT synthesis (Figure 3b). The synthesis method involved introducing various combinations of these substances into a crucible. Nickel wire served as the anode and galvanized iron wire as the cathode. The temperature that the crucible was heated to was 750 °C. The deposition of CNTs with an increasing current density was achieved by progressively raising the current density from 6 mA/cm^2^ to 200 mA/cm^2^ [72].

### 3.5. Chemical Vapor Deposition

**Figure 4:**
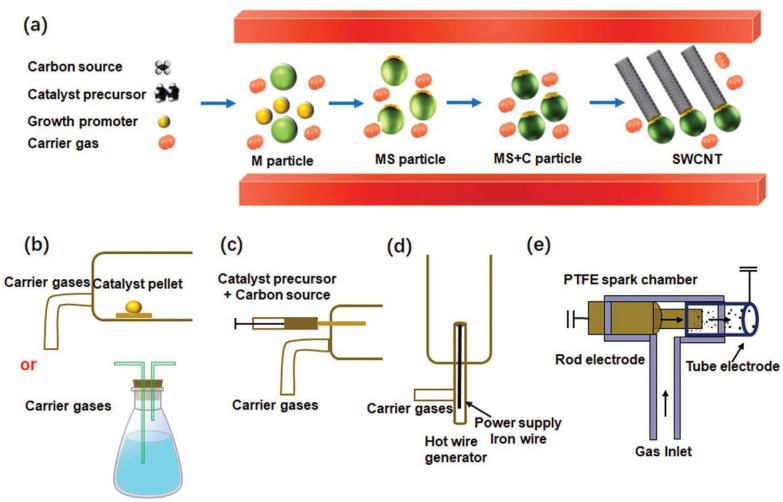
(**a**) Schematic showing the synthesis of CNTs by FCCVD, and (**b**–**e**) various ways of introducing the catalyst into the reactor: supersaturated vapor. Reproduced with permission from [75]. Copyright (2019) John Wiley and Sons.

A suitable catalyst and carbon source are used in the chemical vapor deposition method [73,74]. When it comes to creating carbon nanotubes, this method works like a charm. Chemical vapor deposition produces carbon nanotubes with poor crystallinity, which results in the creation of disordered and misaligned graphene sheaths. TEM (transmission electron microscopy) was used to find this. Inductively coupled plasma vapor deposition is another method that may be used. Plasma from the ICP system is responsible for producing the nanotubes. Through this method, the CNTs display crystallinity up to 500 °C. Two problems exist with using chemical vapor deposition to create carbon nanotubes. The overconsumption of precursors during this process is one concern [73]. The second difference between this method and PECVD is the much higher temperatures used. Nanotubes can only be synthesized at very low temperatures using plasma-assisted CVD. The microstructure creation relies heavily on low-temperature synthesis. In addition, compared to nanotubes synthesized using CVD, those made utilizing PECVD show far lower amounts of contamination. Figure 4a–e illustrates a schematic depicting the production process of carbon nanotubes (CNTs) using the method of fluidized bed chemical vapor deposition (FCCVD). The catalyst precursor (ferrocene), growth promoter (sulfur), and carbon source (ethylene) are continually introduced into a reactor along with a carrier gas. The metallocene catalyst precursor undergoes decomposition at a relatively elevated temperature, resulting in the formation of catalyst nanoparticles by the collision of metal clusters. The carbon precursors undergo decomposition at elevated temperatures, facilitated by the metal nanoparticles acting as catalysts. Concurrently, certain areas of the catalyst nanoparticles experience the formation of hot zones on their surface, maybe resulting from the separation of sulfur. Ultimately, carbon that is fully soaked in specific areas of the molten material solidifies and forms carbon caps, which then elongate into lengthy tubes due to the ongoing provision of carbon. The FCCVD process is distinguished by the synthesis of catalyst nanoparticles and the uninterrupted development of CNTs via the continuous supply of the catalyst precursor and carbon source. Furthermore, the FCCVD technique enables the precise adjustment of various growth parameters, such as the catalyst precursor and its method of introduction into the system, the carbon source, growth temperature, and the type and flow rate of the carrier gas. This level of control allows for the manipulation of the morphology, structure, quality, and yield of CNTs [75].

### 3.6. Catalysis Method

The use of a Co–Mo/SiO_2_ catalyst allowed researchers to create SWCNTs, or single-walled carbon nanotubes. The researchers used a Co–Mo molar ratio of 1:2. The starting materials for the present experiment were ammonium pentamolybdate and cobalt nitrate. Before moving on to the next step, the raw material was calcined at 500 °C. After that, it was placed in a horizontal quartz tube reactor. At 500 °C in an H_2_ atmosphere and 700 °C in a He atmosphere, the precursor was further heated. Carbon monoxide was subsequently used in the production of the single-walled carbon nanotubes [76]. Scientists Liu et al. used catalytic pyrolysis to create MWCNTs, or multi-walled carbon nanotubes [77]. A two-step technique was used to transform polypropylene into MWCNTs. First, catalytic pyrolysis was carried out. Then, a decomposition reaction was carried out. The use of nickel catalysts and HZSM-5 zeolite was part of the synthesis process. Both the first and subsequent stages made use of screw kiln reactors [78].

### 3.7. Plasma-Enhanced Chemical Vapor Deposition

The process of producing nanotubes by the induction of a glow discharge inside the chamber is known as plasma-enhanced chemical vapor deposition [79]. One technique, known as thermal chemical vapor deposition, calls for heating to a certain temperature range, often between 700 and 1000 °C [57]. Despite its many benefits and flexibility, this method is not without its downsides, two specifically. The first issue is that the tubes might not be straight or oriented properly. Furthermore, the substrate material degrades due to the high temperature.

The synthesis of aligned CNTs at low temperatures utilizing PECVD techniques is now the focus of this review [80]. Utilizing plasma-enhanced chemical vapor deposition allows for the manufacture of aligned carbon nanotubes. According to researchers, plasma processing is the method that shows the greatest promise and is the most practical for producing nanotubes at low temperatures [81]. An attractive feature of the PECVD method is the possibility of the production of electron emission sources, such as nanotubes or nanofibers. Microwave amplifiers, parallel electron beam lithography, and displays are just a few of the many applications for these electron emitters [82]. The PECVD technique primarily utilizes plasma. Gaining a solid grasp of plasma’s properties and how glow discharge inductively coupled plasma works is crucial. Table 1 presents the benefits and drawbacks of several synthesis methods for carbon nanotubes (CNTs).

## 4. Perovskite Solar Cell

A key component of inverted PSCs is the perovskite film, which acts as a light-absorbing layer. A compact and homogenous perovskite coating is a necessary condition for preventing unwanted contact between the upper and bottom charge transport layers [87,88].

Furthermore, high-quality films are essential for reducing defect- and energy level-induced nonradiative recombination at the interfaces. Because of these considerations, the VOC and FF values of PSCs are severely limited [89,90]. Improvements in the perovskite film quality have resulted from extensive efforts to optimize the manufacturing processes, fine tune the band gap, and passivate flaws. The Schottky–Queisser limit predicts that photovoltaic solar cells including a perovskite material with an appropriate band gap could achieve power conversion efficiencies greater than 30% [91]. Changing the perovskite’s chemical makeup might do this. A change from perovskites based on methylammonium lead iodide to those based on formamidinium has been implemented in the high-efficiency inverted PSCs. The following types of perovskites are known: mixed-cation perovskites involving FA and Cs, triple-cation perovskites including cesium and FAMA, and mixed-cation perovskites involving FAMA and Cs. Different types of perovskites need different production methods for perovskite films. For inverted PSCs, the current gold standard is a one-step procedure that reliably produces high-quality perovskite films with little waste [92,93]. Due to the substantial impact of manufacturing conditions on the complete conversion of PbI_2_ into perovskite, the traditional two-step solution method, often used for regular PSCs, cannot be applied to inverted perovskite films. The perovskite films produced often have a rough surface, making it difficult to cover the whole perovskite layer with a thin PCBM or C60 layer [94,95]. Consequently, a substantial number of charge carriers are lost due to this insufficient coverage. Vacuum deposition, blade coating, and slot-die coating are examples of scalable procedures often used to manufacture large-scale perovskite solar modules, contrasting with the methods used to create small-scale cells in a laboratory [96,97,98].

## 5. Carbon Nanotube-Based PSCs

Carbon nanotubes and other one-dimensional semiconducting materials with unique architectures have been widely used in perovskite solar cells. Cylindrical carbon nanotubes are a kind of carbon nanomaterial [99]. Their length, diameter, and chirality are just a few of the ways in which these nanotubes may vary in shape and size. They can also have single-walled, double-walled, or multi-walled structures. Because of their unique structure and outstanding properties, such as their high electrical conductivity, huge surface area, excellent transparency to light, and strong resistance to chemical and thermal changes, carbon nanotubes have demonstrated tremendous promise in photovoltaic applications [100,101]. Their unique, extended π-system enables them to provide a highly mobile, direct path for charge transport [102]. Carbon nanotubes have been the subject of a great deal of research for their potential use in perovskite solar cells. In their most basic form, carbon nanotubes are additions that improve charge extraction and stability or a layer that collects charges.

It is the electrical properties of carbon nanotubes that dictate their useful significance in solar cells. Carbon nanotubes made of metal provide a direct path for electrical charges to travel. However, the device’s overall performance is reduced when this specific kind of carbon nanotube is added to the active layer [103]. Semiconducting carbon nanotubes are the material of choice for the photoactive layer because of their ability to reduce charge recombination or contribute electrons to an external circuit.

The first investigation on the incorporation of carbon nanotubes into perovskite solar cells was carried out in [104]. The metal counter electrode in the n-i-p architecture of solar cells was replaced by a laminated CNT network. With an efficiency of 6.9%, the carbon nanotubes doubled as the electrode and the hole-selective layer. The CNT film and transparency allowed it to use its two-sided light power, leading to its efficiency. The lack of charge selectivity and the high resistance of the CNT film are likely reasons for the low efficiency. The power conversion efficiency reached 9.9% due to the improved performance following spiro-OMeTAD incorporation.

By layering a double SWCNT film on LHP and then depositing spiro-OMeTAD, the researchers were able to create a PSC [39]. Without the metal top electrode, the efficiency of the solar cell could reach 15.5%. The device structure, using a scanning electron micrograph, was obtained from a cross-sectional view. Scanning electron microscopy of a cross-section of the solar cell showed long projections, believed to be SWCNTs, detached from the film when the device was cut. By investigating in detail the integration of carbon nanotubes as a charge-conducting layer in perovskite solar cells, the study provides a wealth of information [105].

The inherent mechanical flexibility and outstanding charge transport properties of carbon nanotubes make them very useful. Because of this, they are ideal for use in portable devices, where they may be molded into flexible perovskite solar cells (Table 2). By using a flexible fiber architecture, researchers were able to manufacture photovoltaic solar cells [106]. The configuration positions the light-harvesting perovskite absorber between p-type flexible nanotubes and n-type core wire. Carbon nanotubes sourced from a spinnable array in a dry condition made up the cathode’s outermost layer. Stable even when bent, the fiber-shaped perovskite solar cell had a power conversion efficiency of 3.3%. Although these devices have not yet achieved an efficiency of 10% or more, it is hopeful for a photovoltaic fabric.

Perovskite solar cells using carbon nanotubes, whether single-walled or multi-walled nanotubes, as the hole-selective layer have shown exceptional performance [107,108]. Functionalization improves the carbon nanotubes’ limited ability to disperse in solution, making them a viable alternative for p-type dopants in frequently used hole-transporting materials like spiro-OMeTAD and P_3_HT. The possibility of employing carbon nanotubes as a doping process alternative for spiro-OMeTAD was proven by researchers via the use of a two-layered configuration of CNTs coated with polymers and integrated into an organic matrix [109]. When contrasted with a device that used undoped spiro-OMeTAD, the device demonstrating charge extraction efficiency was much higher. The inability of the nanotubes to form a sufficiently linked percolation network in this configuration is likely responsible for the device’s poor performance compared to a state-of-the-art PSC. Therefore, the fill factor is constrained, since the device’s performance is constrained by series resistance. The absence of enough direct interfacial contact between the LHP absorber and the carbon nanotubes was caused by their dispersion within the spiro-OMeTAD layer. Consequently, they failed miserably in transferring holes from the interface. The carbon nanotubes formed a highly connected network that directly interfaced with the low-halide perovskite layer when spiro-OMeTAD and CNTs were deposited sequentially. The absorber effectively transferred photogenerated holes, allowing for selective hole conveyance.

When carbon nanotubes are used in solar cells, the main advantages are their inherent robustness and longevity. Several approaches have been described for extracting photogenerated charges from perovskite solar cells by using the charge transport properties of carbon nanotubes. An important step toward the technology’s long-term viability would be the substitution of complete carbon nanotube structures synthesized in a solvent for usage in perovskite solar cells’ hole-selective layers and other costly components [110]. This would make the PSCs eco-friendlier and cost effective. If devices using carbon nanotube components can improve their power conversion efficiency, they will be a powerful and advantageous option for dependable, high-performance electronics.

**Table 2:** Properties of CNTs.

Property	Comments	Value	Reference
Intrinsic mobility	Dopant concentration at 1017 cm^−3^ is over a hundred times more than silicon’s at 300 K	At ambient temperature for individual CNTs: above 10^5^ cm^2^/Vs	[111]
Surface area	More than the 1200 m^2^/g value for activated carbon	1600 m^2^/g	[112]
On/off current ratios	Temperature one thousand times greater than that of a bilayer structure	Over 10^5^	[113,114]
Thermal conductivity	Diamonds’ is about 1500 Wm^−1^ K^−1^ greater	Reaching 3500 Wm^−1^ K^−1^.	[115]
Fracture stress	Size of the steel wires is about 50 times smaller after density normalization	50 GPa	[116]
Electrical conductivity	Comparable to that of mercury and other metals	104 × 10^6^ S cm^−1^	[117]
Free carrier concentration	Lower compared to graphene (~1020 cm^−3^) and the majority of metals (such as silver, which has ~1022 cm^−3^)	~1017 cm^−3^	[118]
Current carrying capacity	Copper has a 1000-fold lower value	Surpassing 109 A cm^−2^ for a single CNT	[119]
Young’s modulus	Of a single-crystal diamond at ambient temperature	1–2 TPa	[120]

### 5.1. Carbon Nanotubes as Hole Transport Layers

Using its p-type properties, the hole transport layer blocks the flow of photogenerated electrons, and at the same time, attracts and retains holes, making it easier for them to go from the active layer to the electrode [121,122,123]. As a result, a good HTL will have well-aligned energy levels, great thermal stability, and high hole mobility. When it comes to p-i-n and n-i-p perovskite devices, the Spiro-OMeTAD and PEDOT:PSS are often used as HTLs, or highly efficient hole transport layers, respectively. Several aspects have prompted researchers to look for alternative hole transport layers (HTLs); however, they are either too pricey, unstable, or too hygroscopic [100,124,125]. Due to their excellent hydrophobic qualities, low cost, and exceptional stability, carbon nanotubes have lately been acknowledged as very promising hole transport layers in perovskite devices.

A carbon nanotube sheet coated with poly(methylmethacrylate) was used in an experiment carried out by researchers [126]. The efficiency of the perovskite solar cells was 5.82% when this film was used as an HTL. The researchers found that the contact resistance between the perovskites and CNTs was reduced as a result of the increase in contact due to the shrinkage of PMMA. Compared to the PSC that used Spiro-OMeTAD as the hole transport layer, the device’s VOC of 1.45 V was much higher. Despite this, the CNT layer exhibited little charge carrier selectivity and was widely believed to be a conductor [127]. A viable solution to this issue is the creation of hybrid hole transport layers by incorporating hole transport materials into carbon nanotube films. The double hole transport layers are fabricated in n-i-p devices employing single-walled carbon nanotubes coated with poly(3-hexylthiophene) and polymethyl methacrylate [100]. Functionalizing P3HT increased the p-type features and changed the electrical properties of SWCNTs. At the same time, PMMA was applied to the P3HT/SWCNTs’ surface to prevent oxygen and moisture from reaching the perovskite layer by acting as an encapsulant. A final efficiency of 14.2% and enhanced stability were shown by the devices, as well as the formation of a hole-conducting protective layer. This layer was created utilizing a thin film that included SWCNT, GO, and PMMA. The improved hole-selective transport allowed the mesoscopic n-i-p structured device to achieve a PCE of 11.7%, showing the capabilities of single-walled carbon nanotubes/graphene oxide. Devices employing Spiro-OMeTAD had a PCE similar to this. Furthermore, by honing the method of perovskite layer fabrication, the power conversion efficiency of the GO/SWCNT/PMMA device could be raised to 13.3%.

The insulating properties of PMMA prevent it from improving the hole transport capacity of CNTs, but its protective and interfacial-enhancing properties may make PSCs more stable and efficient. A possible solution to improve devices’ photovoltaic performance is to use hole-conductive polymers instead of PMMA. The n-i-p devices developed made use of a double hole transport layer [128]. A mix of P3HT/SWCNTs and undoped Spiro-OMeTAD made up the HTL. In comparison to the perovskite solar cell that used just undoped Spiro-OMeTAD, which had a PCE of 6.8%, the obtained PCE of 15.4% was much higher. An increase in the solar efficiency was seen as a result of the inclusion of a hole transport bilayer including single-walled carbon nanotubes, which accelerated the charge extraction process. To improve the power conversion efficiency to 18.8%, FA_0.83_MA_0.17_Pb(I_0.83_Br_0.17_)_3_ was used as the light absorber layer and SnO_2_ as the electron-accepting layer in the next study. When compared to devices that used Li-doped Spiro-OMeTAD as HTLs or devices that used undoped Spiro-OMeTAD, this one performed better. To efficiently disperse SWCNTs and MWCNTs in organic solvents, researchers used ethylene vinyl acetate, a non-conjugated polymer that is both inexpensive and effective [129]. The result of this procedure was conductive films made of high-quality, EVA-functionalized SWCNTs and MWCNTs. The double hole transport layers of perovskite solar cells were then modified to employ two films instead of P3HT/CNTs, leading to PCEs of 17.1% and 16.8%, respectively.

**Figure 5:**
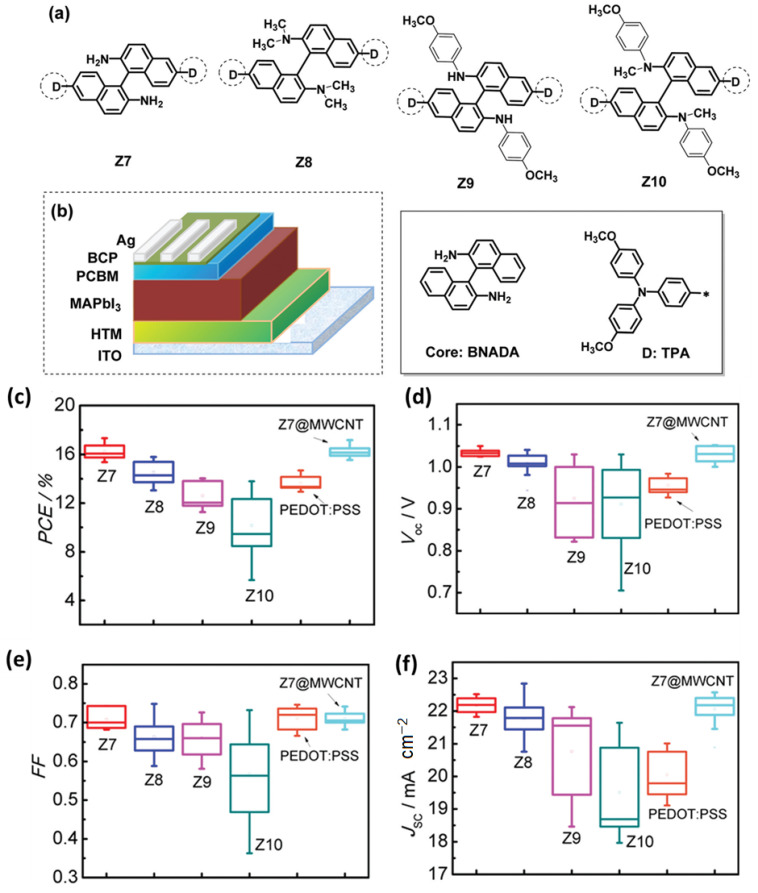
(**a**) HTM chemical structures, (**b**) design of inverted planar perovskite solar cells, (**c**) PCE, (**d**) V_oc_, and (**e**,**f**) J_sc_ of device. Reproduced with permission from [131]. Copyright (2019) Royal Society of Chemistry.

Perovskite solar cells are very efficient and stable when using a mix of carbon nanotubes and other hole transport materials in addition to the double HTLs already discussed. To generate novel hybrid hole transport layers, researchers mixed PEDOT:PSS with single-walled carbon nanotubes [130]. Novel hole transport molecules, based on binaphthylamine, were developed and synthesized, each having distinct side chains. The materials developed have similar optoelectronic capabilities and molecular structures but differ in terms of their film properties (Figure 5a,b). Two types of hole transport molecules, optimized with amine and methyl units, were attached to multi-walled carbon nanotubes through physisorption. These functionalized carbon nanotubes were then utilized in the production of inverted perovskite solar cells. An unencapsulated device using a hole-transporting material integrated with multi-walled carbon nanotubes achieved a power conversion efficiency of 17.17%, which was similar to the efficiency of the original hole transport material (17.32%). The HTM@MWCNTs device achieved a maximum PCE of 17.17% (Figure 5c), a V_oc_ of 1.052 V (Figure 5d), and an FF of 0.72 (Figure 5e), with a J_sc_ of 22.57 mA/cm^2^ (Figure 5f). Furthermore, the former demonstrates superior gadget stability compared to the latter. The use of carbon nanotubes in hole transport materials offers a novel approach to enhancing the stability of inverted perovskite solar cells [131]. The outcome was the establishment of a strong chain structure that seemed to include minute molecules. The p-i-n PSC without encapsulation outperformed the PSC with a PEDOT:PSS-based hole transport layer in terms of its stability and efficiency, reaching 17.2% compared to 14.69%. It was also discovered that a hybrid material made of nickel oxide and carbon nanotubes made for an excellent hole transport layer. A hybrid hole transport layer (HTL) was created by adding carbon nanotubes to a solution that already included a nickel oxide precursor [132]. An increased conductivity and improved hole extraction were observed in this hybrid HTL, which retained the optical and surface morphological properties of NiO_x_. The power conversion efficiency of the enhanced p-i-n planar device was 16.9%. The results indicate that the hybrid HTLs might be useful in planning PSCs that are stable, efficient, and processed at low temperatures.

Perovskite solar cells may benefit from hole transport layers made of carbon nanotubes, as shown before. Increasing the photocurrent and fill factor via improved conductivities and hole mobilities is essential for producing PSCs with a high efficiency. It should also be considered that these unconventional hole transport layers have a lower hole selectivity than conventional ones. Perovskite solar cells that use carbon nanotubes would be more efficient if carbon nanomaterials were functionalized and hybrid films were made using traditional hole transport materials.

### 5.2. Carbon Nanotubes as Perovskite Layer Additives

A great variety of grain boundaries, including several defects/traps, are common in perovskite films produced using a solution. It causes a great deal of charge recombination. Perovskite devices lose efficiency and stability due to these defects, which significantly affect internal ion mobility [133,134]. For this reason, producing high-performance PSCs requires exact control over the perovskite layer structure and crystallinity [135]. The grain sizes and defect density of perovskites have been improved using a variety of approaches. Some of these include the use of inorganic and organic salts [136,137,138,139] in solution-based methods, as well as ionic liquids [140,141,142] and Lewis acids/bases [143,144]. Since carbon nanotubes have a unique structure, outstanding optoelectronic characteristics, and are chemically inert, researchers are interested in incorporating them to improve the functionality of the device by way of the perovskite layer.

To facilitate the transfer of charges between the perovskite grains, Cheng et al. [94] integrated multi-walled carbon nanotubes into perovskite layers. Additionally, MWCNTs improved the carbon electrodes’ hole-collecting efficiency. Compared to PSCs devoid of multi-walled carbon nanotubes, the artificially generated perovskite solar cell achieved a PCE of 11.6%, showing an increase of almost 15%. In their study, the researchers looked at how perovskite crystallization was affected by functionalized multi-walled carbon nanotubes. For the perovskite film, the scientists found that sulfonate carbon nanotubes could both fill up the spaces between grains and make the micron-sized grains larger [103]. Those results could not have been achieved with the initial carbon nanotubes, however. Perovskite crystals are formed when methylammonium iodide molecules aggregate around s-CNTs as a result of the interactions between sulfonic acid and PbI_2_. If the material were to undergo further heat treatment, perovskite grains would be more effectively formed, with the s-CNTs remaining at the grain boundaries. The power conversion efficiency of the artificially generated perovskite solar cell utilizing single-walled carbon nanotubes was 15.1%, which was higher than the performance of the PSC employing untreated carbon nanotubes, which was 10.3%. The perovskites’ crystallinity and orientation were controlled by adding amino-functionalized carbon nanotubes and methylammonium chloride [145]. Crystal grain formation was aided by the introduction of MACl, which created the intermediate phase. At the same time, the inclusion of CNT-NH_2_ caused the grain sizes to grow. This could be because there were more opportunities for heterogeneous nucleation to produce perovskites due to the strong affinity between the -NH_2_ functional groups and Pb^2+^. In addition, perovskites may have their orientation affected by reductions in the surface energies of (110) crystal facets brought about by the presence of CNT-NH_2_ functional groups. The grain size, crystal orientation, and crystallinity were all improved in the MA_0.85_FA_0.15_PbI_3_ film when it was mixed with MACl and CNT-NH_2_. Consequently, it showed little recombination and an effective charge extraction and transport. Therefore, the device that used this exceptional perovskite showed exceptional consistency and longevity, and it obtained a power conversion efficiency of 21.05%. According to recent research, CNT-NH_2_ has the potential to improve the development of crystal grains in perovskite films by acting as a growth template [146]. It may also speed up the extraction and transport of charges by acting as a carrier transfer route.

**Figure 6:**
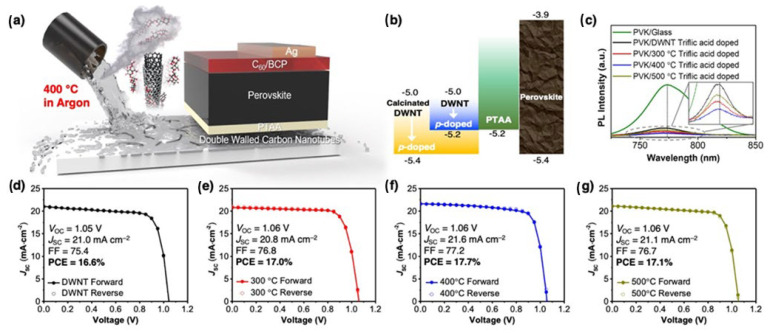
(**a**) A three-dimensional rendering of a PSC device using a transparent electrode made of calcinated DWNT. (**b**) Interfacial energy diagram of DWNTs, PTAA, and perovskites. (**c**) PL quenching of the perovskite layer (PVK) on DWNT doped with triflic acid (black), DVNT doped with triflic acid (red), DWNT doped with triflic acid (blue), and DWNT doped with triflic acid (khaki) after being calcinated at 300 °C, 400 °C, and 500 °C, respectively. The photovoltaic parameters of the PSCs based on (**d**) triflic acid-doped pristine DWNT, (**e**) triflic acid-doped 300 °C-calcinated DWNT, (**f**) triflic acid-doped 400 °C-calcinated DWNT, and (**g**) triflic acid-doped 500 °C-calcinated DWNT were determined by observing the J-V forward and reverse bias curves under AM 1.5 G one-sun illumination. Reproduced with permission from [147]. Copyright (2021) John Wiley and Sons.

The fabrication of inverted-type PSCs was carried out to verify the device applicability and increased doping impact of the surfactant-removed DWNT transparent electrodes. After a 10 min heat treatment in Ar, a glass substrate was slot-die coated with DWNT solution. A configuration of DWNT/poly(triaryl amine) (PTAA) [35 nm] was used to manufacture the remaining layers. 2,7-fluorene containing 9,9-dioctylfluorene bonded to an alkyl group as a polymer PFN-P2 [<10 nm]/MA_0.6_FA_0.4_PbI_2.9_Br_0.1_ [450 nm]/C60 [20 nm] (dibromide) Figure 6a shows the chemical formula for the compound/bathocuproine (BCP) [6 nm]/Ag [70 nm]. Figure 6b indicates that the valence band of the perovskite layer, not the PTAA layer, is more in line with the Fermi level of the triflic acid-doped, surfactant-removed DWNT electrode. In contrast, the pristine DWNT electrode doped with triflic acid has a Fermi level that is in good alignment with the PTAA layer’s valence band, because the p-doping is rather modest. Figure 6c shows that the calcinated DWNTs produce a more effective charge transfer and decreased trap states, respectively, Eliminating the surfactants improves the charge transfer, suggesting that they behave as trap states. The quenching characteristics are much lower in DWNTs calcinated at 500 °C, which is an intriguing finding. Figure 6d–f shows that the FF is the major source of the performance variation among the manufactured devices, all of which display a high performance. Nevertheless, the disparity did not meet our expectations. Electronics built with p-doped DWNT electrodes calcinated at 400 °C exhibited, as predicted, the lowest series resistance, as seen in Figure 6g, as a result of the most significant p-doping impact. Because of this, the maximum FF was 77.2. When triflic acid was doped into DWNT films, their conductivity was enhanced. Although the surfactant-wrapped DWNT films had a conductivity gain of 31.9%, the DWNT films with the surfactants removed had a conductivity increase of 59.7%. The inverted-type perovskite solar cells achieved a power conversion efficiency of 17.7 percent without hysteresis by using p-doped, transparent electrodes that had been solution processed after the surfactant had been removed. The best efficiency ever attained for carbon nanotube electrode-based perovskite solar cells and solution-processable transparent electrode-based solar cells is a result of their study, which improved the use of DWNTs in transparent conductors [147].

The making of appropriate surfactants that can increase their solubility is essential for solving the issue. Maruyama’s group demonstrated that s-SWCNTs, which are semiconducting nanotubes, can be both charge transport channels and templates for perovskite crystal formation. As a result, the PSCs’ power conversion efficiency rose from 18.1% to 19.5% [46]. To make single-walled carbon nanotubes more evenly dispersed in water, the researchers utilized the surfactant sodium deoxycholate. At the same time, grain boundaries might be rendered inactive by the Lewis adducts created by the carbonyl groups found in DOC. The results of the fast Fourier transform and TEM imaging analysis confirmed the existence of perovskite grains on the single-walled carbon nanotube. Even though the performance was improved, the device’s fill factor was limited by DOC’s insulating properties. To continue with the study, a novel surfactant known as 4,6-di(anthracen-9-yl)-1,3-phenylene bis(dimethylcarbamate) was utilized instead of DOC. DPB has the required energy level and an outstanding mobility. The researchers found that s-SWCNT was more soluble in N, N-dimethylformamide, a solvent often used for perovskite precursors, when DPB was added. The bigger perovskite crystals, improved passivation, and less charge trapping were all made possible by the DPB-attached single-walled carbon nanotubes. An efficiency of 20.7% was therefore attained, which was higher than that of the PSC, using SWCNTs in conjunction with DOC.

The lead perovskites used in most high-performance PSCs are commercially unfeasible due to the risks associated with using them. One major problem that might cause PSCs to degrade is lead leaking from the perovskite layers [148]. Researchers have tried to make perovskite solar cells (PSCs) out of tin instead of lead, but the results are much less efficient [149,150]. Therefore, managing the discharge of lead is crucial. A perovskite film was enhanced in recent work by Wang et al. with the addition of carbon nanotubes that were covalently functionalized with poly(acrylic acid). With this enhancement, lead leakage was significantly reduced, and the power conversion efficiency reached an impressive 21.8%. High concentrations of carboxyl groups were observed in the research when a thick coating of PAA was applied to the surface of the CNT framework. As seen in these groups, these were crucial in promoting perovskite formation and efficiently immobilizing the Pb ions. In the perovskite layer, in addition to serving as charge transport routes, CNT-PAA may reduce unwanted defect states. The effect of PAA’s molecular weight on the PSC’s photovoltaic performance was studied by combining PAA with CNTs of varying weights. These combinations were named after the 22.4% and 35.1% PAA weight contents, respectively. The photovoltaic parameters were better with the CNT-PAA-L addition than with the CNT-PAA-S additive. One possible explanation for this enhancement is that the CNT-PAA-L additive has more functional sites on its longer side chains [151]. Using a flame atomic absorption spectrometer, the lead concentration was analyzed to quantify the devices’ lead leakage. As compared to devices containing CNT-PAA-S (4.4 ppm) and CNT-PAA-L (2.9 ppm), the control device leaked 9.5 ppm of Pb^2+^ after 600 min, a substantially higher concentration. This finding proved that lead release is significantly inhibited when CNT-PAA is present. The sequestration efficacy of the device containing CNT-PAA-L was around 70%. The production of insoluble CNT-PAA-Pb compounds was caused by the significant chelation of CNT-PAA with Pb^2+^, which allowed for the efficient binding of lead.

Crystals may be shaped in the precursor solution using functionalized carbon nanotubes, as discussed in this section. This opens the door to the fabrication of large-scale or highly oriented-perovskite films. Additionally, functionalized carbon nanotubes contribute to the reduction of charge traps, passivation of defects, and suppression of lead leakage via their abundant functional groups. Additionally, the charge transfer efficiency is improved when carbon nanotubes are present at the grain boundaries of perovskites. The creation of efficient, stable, and ecologically friendly photovoltaic solar cells can be made possible by these effects.

### 5.3. Carbon Nanotubes in Electron Transport Layers

ETLs help to prevent holes from moving while also facilitating the extraction of photogenerated electrons from active layers to TEs or CEs effectively [152]. Therefore, a master ETL has to have a lot of features, such as a high degree of chemical stability, great electron mobility, and the right amount of energy to efficiently capture electrons [153,154]. To be specific, for n-i-p PSCs to work, the ETL’s optical transmittance has to be high so that enough light can reach the perovskite light absorber. In conventional perovskite solar cells, the electron transport layer often contains n-type semiconducting metal oxide nanoparticles, including TiO_2_, ZnO, and SnO_2_. Nevertheless, due to many grain boundaries and unexpected transport paths, charge recombination is often accelerated when disordered nanoparticles are present in ETLs [155]. Nanoparticle films for flexible devices manufactured on plastic substrates are also limited in their capacity to raise the annealing temperature. Several methods have been shown to improve the efficiency of ETLs. Integrating carbon nanomaterials with metal oxide nanoparticles is a reliable approach that enhances electron extraction and transportation in perovskite devices [156,157,158].

Excellent electron motilities are a property of one-dimensional materials, such as carbon nanotubes. As a result, they might act as electroconductive channels inside films of nanoparticles [159]. Although carbon nanotubes display a p-type behavior when oxygen molecules are present on their surfaces, previous studies have shown that CNTs have the potential to make TiO_2_ nanofibers more electron mobile and directionally sensitive [160]. The n-i-p perovskite solar cell devices are made by using porous titanium dioxide sheets and single-walled carbon nanotubes. The short-circuit current density increased from 19.0 to 21.6 milliamperes per square centimeter (mA cm^−2^) due to the enhanced electron mobility and reduced charge carrier loss caused by the incorporation of 0.1 weight percent of single-walled carbon nanotubes. Additionally, a band energy alignment was formed due to the displacement of titanium dioxide’s conduction band minimum, which was generated by the insertion of SWCNTs. The open-circuit voltage rose from 0.986 V to 1.002 V as a result of this alignment. The power conversion efficiency came out at 16.11% as a result. Not only that, but SWCNTs may improve the current–voltage properties of perovskite solar cells while decreasing hysteresis. However, when the concentration of SWCNTs was increased even higher, a notable charge recombination event was seen because of the existence of many junctions that served as recombination foci. Also, how exactly these composite electron transport layers made of metallic SWCNTs and semiconducting SWCNTs work to boost perovskite solar cells’ efficiency is still a mystery. Researchers produced semiconducting SWCNTs and metallic SWCNTs and inserted them into titanium dioxide electron transport layers to investigate the effects of various types of SWCNTs on the electronic characteristics [161]. Among the PSCs, the one with the best efficiency (19.4%) was made using a 2:1 ratio of semiconducting to metallic single-walled carbon nanotubes. Power conversion efficiencies of 17.04%, 18.10%, 17.77%, 17.21%, and 18.09% were exhibited by devices that did not contain single-walled carbon nanotubes, devices that contained only semiconducting SWCNTs, devices with 1:2 ratios of s-SWCNTs to m-SWCNTs, and devices with a 1:1 ratio of s-SWCNTs to m-SWCNTs. Device power conversion efficiencies are undeniably enhanced by the use of single-walled carbon nanotubes. The device that had just m-SWCNTs, on the other hand, had a lower JSC than the other PSCs. This could be because of the fast-paced charge recombination around the m-SWCNTs’ Fermi level and the inadequate intrinsic potentials of m-SWCNT/TiO_2_ interfaces. To study various conductivities of single-walled carbon nanotubes, time-resolved photoluminescence characterizations were carried. In line with earlier findings in the J-V curves, the results showed that the electron transport layer with a 2:1 ratio of s-:m-SWCNTs/TiO_2_ produced the fastest decay time. It has been shown via density functional theory simulations that excited electrons inside SWCNTs may be able to move to the surface of TiO_2_. In addition, it was shown that metallic single-walled carbon nanotubes may improve this transfer process when mixed with other forms of SWCNTs. Surprisingly, the incorporation of m-SWCNTs into the mixed single-walled nanotubes significantly slowed down the degradation rate, making the devices last longer, because they are less sensitive to environmental influences. These results indicate that perovskite devices made from a mixture of single-walled carbon nanotubes would be efficient and long lived. To study the performance of perovskite solar cell interfaces, researchers have used various spectroscopic techniques such as photoluminescence, femtosecond transient absorption spectroscopy, and time-correlated single-photon counting to evaluate the composition and charge transfer electron transport layer and perovskite layers [162]. It was found that the quenching efficiency of TiO_2_/MAPbI_3_ was higher than that of CNT-TiO_2_/MAPbI_3_ using PL spectroscopy. This indicates that the charge transfer from MAPbI_3_ to TiO_2_ is more efficient than CNT-TiO_2_, which is contrary to their previous findings. This was due to the use of a separate MAPbI_3_ coating technique in the two studies. Time-correlated single-photon counting spectra were created and analyzed to determine the core component (τ2) and surface component (τ1). The surface component represents trap-assisted processes, while the core component represents free-charge recombination [10]. The decay periods for the τ1 value were 0.58 ns for TiO_2_/MAPbI_3_ and 0.73 ns for CNT-TiO_2_/MAPbI_3_. This suggests that the addition of CNTs did not enhance the charge transfer, which aligns with the results obtained from the PL spectra. The τ2 value for the CNT-TiO_2_/MAPbI_3_ was 97 ns, which was more than the τ2 value of the TiO_2_/MAPbI_3_. The extended lifespan (τ2) demonstrated a reduced rate of bimolecular recombination in the MAPbI_3_/CNT-TiO_2_ system. The fs-TAS and decay kinetics dynamics of TiO_2_-MAPbI_3_ and CNT-TiO_2_/MAPbI_3_ layers were compared. The decay time for TiO_2_/MAPbI_3_ (5.7 ns) was shorter than that for CNT/MAPbI_3_ (8.6 ns), providing further evidence of a faster charge transfer for the former. In summary, carbon nanotubes did not improve the transfer of charges at the interface, but researchers successfully inhibited the presence of trap states, resulting in a reduction in recombination in the device. Remarkably, the existence of carbon nanotubes increased the resistance to recombination while reducing the capacitance related to chemical reactions. With a power conversion efficiency of 20.4%, the built-in n-i-p device using the CNT-TiO_2_ composite ETL outperformed the control device without CNTs, which had a PCE of 18.4%. Electron transport layers based on titanium dioxide have both SW and MW carbon nanotubes. Using multi-walled carbon nanotubes as additives in titanium dioxide electron transport layers to alter the behavior of n-i-p devices resulted in an efficiency of 21.4% [163]. The addition of MWCNTs caused the ETL Fermi level to change from −4.27 eV to −4.03 eV. This resulted in a higher energy level at the interface between MWCNT-TiO_2_ and the perovskite, causing an increase in the VOC from 0.845 V to 1.085 V. In addition, linking multi-walled carbon nanotubes led to the formation and improvement of larger grains. A high crystallinity was observed in the perovskite film, which improved its ability to absorb light. Researchers used MWCNT–graphene–TiO_2_ hybrid films as electron transport layers to fabricate n-i-p perovskite solar cells [164]. The CNT–graphene–TiO_2_-based device exhibited a higher JSC (short-circuit current density) of 24.8 mA cm^−2^ and a higher PCE (power conversion efficiency) of 13.97% compared to devices using bare TiO_2_, CNT-TiO_2_, and graphene–TiO_2_ electron transport layers. The results were attributed to the synergistic effect of the excellent electron-withdrawing ability of multi-walled carbon nanotubes and the extensive specific surface areas provided by graphene sheets, as shown in studies on photoluminescence and photovoltage decay. Hence, the coexistence of carbon nanotubes and graphene, rather than their separate use, in an electron transport layer system could enhance charge collection in perovskite solar cells to a greater extent.

**Figure 7:**
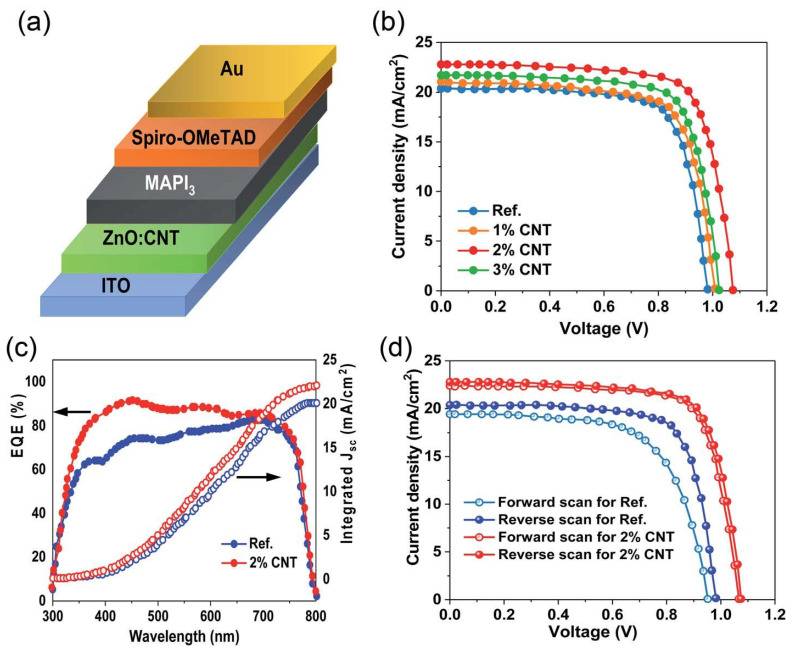
(**a**) A schematic showing how the gadgets built for this research are structured. (**b**) PSC J-V graphs. (**c**) Electron spin resonance spectra of doped and undoped PSCs. (**d**) Effects of hysteresis on PSCs containing pure ZnO and ZnO with 2% CNT. Reproduced with permission from [167]. Copyright (2019) Royal Society of Chemistry.

Zinc oxide and tin dioxide are also efficient electron transport layers in perovskite solar cells. However, these devices exhibit hysteresis effects and instability due to defects at the interface between the perovskite and ETL, as well as imbalanced charge transfer [165,166]. To tackle these difficulties, researchers used carbon nanotubes in the zinc oxide electron transport layer to fabricate n-i-p perovskite solar cells [167]. It was discovered that the deterioration of perovskite films was caused by specific positively charged ions from hydroxyl groups on the surface of ZnO. Carbon nanotubes may efficiently modify the surface of zinc oxide and shield perovskite materials from hydroxyl agents, leading to an enhanced stability and reduced J-V hysteresis. Furthermore, the use of carbon nanotubes resulted in the attainment of a superior perovskite film quality and improved charge extraction capabilities. The utilization of ZnO ETLs, as depicted in Figure 7a, involved the adoption of a conventional architecture (n-i-p). The evaluation of the functionality of the PSCs was conducted by obtaining current density–voltage (J-V) curves, as depicted in Figure 7b. The experiment revealed that the introduction of a minor quantity of carbon nanotube (CNT) doping into zinc oxide (ZnO) led to an increase in both the short-current density (J_sc_) and open-circuit voltage (V_oc_) when compared to the device produced using pure ZnO (reference). The correlation between the integrated current density, which corresponds to the external quantum efficiency (EQE) values for the reference and champion perovskite solar cells (PSCs), and the scanning quantum efficiency (J_sc_) is depicted in Figure 7c. Hysteresis behavior of the J-V curve is commonly observed in the n-i-p architecture. The hysteresis was examined through the measurement of J-V curves obtained from the forward and reverse scans of both the reference and champion devices. According to the data shown in Figure 7d, the champion device exhibited less hysteresis in comparison to the reference device. Hence, it can be inferred that the introduction of carbon nanotubes (CNTs) into zinc oxide (ZnO) results in the mitigation of charge carrier traps, thereby successfully diminishing the hysteresis phenomenon. Consequently, the PCE increased from 15.05% to 18.79% [167].

Scientists have developed n-i-p devices with an improved efficiency and no hysteresis using hybrid electron transport layers made of carbon nanotubes and silver oxide [168]. The carbon nanotubes underwent the first modification via oxidation procedures before being combined with SnCl_4_. The combination of solutions was applied onto the ITO substrate, resulting in the formation of the CNT-SnO_2_ hybrid electron transport layer during annealing. The study revealed that carbon nanotubes successfully improved the efficiency of electron extraction, as shown by photoluminescence and electrochemical impedance spectroscopy tests, by lowering SnO_2_’s resistance and trap-state density. The device incorporating carbon nanotubes achieved a power conversion efficiency of 20.33%, surpassing the efficiency of the referenced perovskite solar cell, which had an efficiency of 17.90%.

The results indicated that carbon nanotubes can serve as efficient additives in metal oxide electron transport layers to improve electron conduction and decrease defect densities. However, only a small number of studies directly utilize carbon nanotube films, or modified CNT films, as electron transport layers in perovskite solar cells. This is likely because the carbon nanotube film, as prepared, exhibits a p-type characteristic in the presence of oxygen molecules. Hence, the creation of reliable n-type carbon nanotube films is anticipated for implementing CNT-based electron transport layers in perovskite solar cells.

### 5.4. CNTs as Interlayers

Many interfaces are formed by the usual device structure of a perovskite solar cell, which is composed of many layers organized sequentially. Vapors, hanging bonds, and a misalignment of energy levels are common issues seen at these interfaces. Because of these defects, PSCs may degrade irreversibly, have J-V hysteresis, and have a poor solar performance [169,170]. To improve the charge transmission and decrease interfacial recombination, perovskite materials and HTLs or electrodes often use interlayers [171,172]. Furthermore, the interlayer greatly enhances the stability, which is beneficial for PSCs’ commercial use [173].

To improve the interfaces in PSCs, several interfacial materials have been used, such as polymers, carbon nanomaterials, and metal oxides. Due to their exceptional electrical properties, large specific surface area, and special one-dimensional structure, carbon nanotubes stand out among these interlayers, showing tremendous promise. In perovskite solar cells, researchers found that s-SWCNTs, which are highly enriched single-walled carbon nanotubes [174], can improve hole extraction and reduce recombination and reverse charge transfer. The researchers successfully created long-lasting charge separation by placing MAPbI_3_ and Spiro-OMeTAD on a single-walled carbon nanotube membrane. Using a 5 nm thick (6.5) s-SWCNT interlayer improved the photovoltaic performance of the PSC, according to the study. The main reason for the increase was a significant improvement in the duty cycle and short-circuit current density, or JSC. The researchers proposed the idea that D-MWCNTs, which are defective multi-walled carbon nanotubes, could potentially change the charge transfer rate at the interface between the hydrogen transport layer and the graphene electrodes [175]. Interactions of electrostatic dipole moments between Spiro-OMeTAD and the oxygen groups of D-MWCNTs modified the energy level of the hole transport layer. A faster charge transfer was the outcome of this change, which was made possible by one-dimensional channels and electron redistributions. The interface interaction between the micro-sized graphene and D-MWCNTs allowed for the simultaneous construction of flawless connections between HTLs and carbon electrodes. Not only was the gadget quite stable while functioning, but its power conversion efficiency was an astounding 22.07%.

As locations for the crystal formation of perovskites, functionalized carbon nanotubes have better surface properties. The abundance of functional groups on CNTs improves the interface contact with perovskites, which is the reason for this. The creation of a thick layer of carbon nanotubes on top of the perovskite layer using a spin-coating deposition process while avoiding breakdown is a substantial challenge. A straightforward and efficient method for coating perovskites in n-i-p devices with octadecylamine-functionalized single-walled carbon nanotubes was developed by researchers [176]. The ODA-SWCNTs floated beautifully in chlorobenzene and showed improved hydrophobic characteristics. A dramatic increase in the grain size was seen when ODA-SWCNTs were deposited onto the moist perovskite surface. Carbonyl groups on ODA-SWCNTs were thought to be responsible for the noticeable structural change in the perovskite film. Perovskite grains form and grow on SWCNT surfaces due to the strong attraction of these groups to Pb^2+^ ions. The network of SWCNTs may also slow the evaporation rate of polar solvents like dimethylsulfoxide and dimethylformamide. Because of the reduction in evaporation rates, the density at which perovskite grains form is reduced, and larger grains are formed. The study further shows that during annealing, a condition of dynamic near equilibrium between SWCNTs and perovskite may result from the presence of solvent vapors trapped within the perovskite layers. This led to the vertical growth of perovskite grains on the material’s surface, which were micrometers in size. The researchers used ultrafast transient absorbance measurements to examine the photogenerated charge dynamics, specifically the charge transfers in the perovskite layer, with and without the ODA-SWCNT sheet. A faster charge transfer and longer carrier lifespan were the outcomes of using ODA-SWCNTs. This might be because the perovskite layers have smaller grains and less distinct grain boundaries. Thanks to their advantageous shape, perovskite solar cells were able to significantly improve their short-circuit current and eliminate hysteresis. In terms of the power conversion efficiency, devices that used (FA_0.83_MA_0.17_)_0.95_Cs_0.05_Pb(I_0.83_Br_0.17_)_3_ as their light-absorbing material reached the maximum value of 16.1%. Because ODA-SWCNTs are more hydrophobic, the device was also very stable in environments with a high humidity. One significant way to improve device performance is to create compound interlayers based on nanocarbon that has large specific surface areas and outstanding conductivities. A core-shell hybrid made of carbon nanotubes and graphene was prepared by researchers [177]. This was accomplished by using plasma-enhanced CVD to produce graphene on CNT cores. To bridge the gap between Spiro-OMeTAD and Au, these hybrids were subsequently used. The hybrid material’s remarkable thermal stability is a result of its ability to physically retain Spiro-OMeTAD molecules, which result from the π–π interactions between nanocarbons and Spiro-OMeTAD. Surprisingly, the CNT@G/Spiro-OMeTAD layer had a dual purpose: it effectively blocked the migration of iodide in perovskites and also prevented moisture from penetrating the material from humid environments. Because the nanohybrid interlayers are more electrically conductive, electron deficits, or holes, might be more effectively transferred by connecting Spiro-OMeTAD with the CNT@G nanohybrid. This was followed by the quick transfer of these holes to Au. Incorporating CNT@G into the device resulted in a 19.56% power conversion efficiency, which was accompanied by outstanding water and thermal stability.

**Figure 8:**
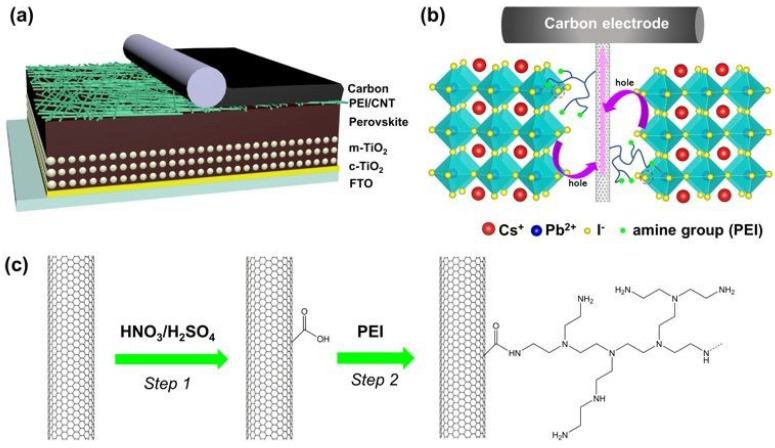
(**a**) The design of the device at the perovskite/carbon electrode interface for C-PSCs with implanted PEI/CNT bridging. (**b**) A schematic showing the charge transfer mechanism from perovskite to carbon electrode using PEI/CNT as the bridge, and perovskite surface trap state passivation molecules also appear in the schematic. (**c**) Artificially prepared PEI-functionalized carbon nanotubes. Reproduced with permission from [180]. Copyright (2019) Royal Society of Chemistry.

Notable features, such as great stability and cost, are shown by carbon-based PSCs that do not include halogenated tin–lead. However, the doctor-blading process is often used to make most paintable carbon pastes on the perovskite layer. Because of the high number of cracks and holes that form at the perovskite/carbon interfaces produced by this technique, they are often of a poor quality. To solve this issue, researchers devised a method that involved spinning a perovskite precursor solution containing a MWCNT dispersion [178]. This enabled the MWCNTs to penetrate both the perovskite material and the carbon electrodes. Hence, MWCNTs (multi-walled carbon nanotubes) may serve as channels for the transmission of electrical charges. An investigation of the hole transmission from perovskites to carbon electrodes was conducted using the photoluminescence spectra. The addition of carbon nanotubes significantly reduced the photoluminescence intensity. In comparison to the post-MWCNTs created using the spin-coating method, the perovskite layer produced by dripping MWCNTs showed a more notable decrease in the PL intensity. This provides further evidence that MWCNTs have a useful function in improving the conduction of holes. In addition, the EIS Bode graphs showed that the device’s characteristic frequency peak shifted towards the lowest frequency when MWCNT dripping was applied. The carriers have a longer lifespan, as this trend suggests. The power conversion efficiency reached 13.57% as a result of the infiltrating MWCNTs improving the perovskite–carbon link, which in turn boosted the VOC and FF values. Researchers enhanced the perovskite–carbon interface using single-walled carbon nanotubes [179]. The researchers opted for MAPbI_3_, a substance that is often used, as the light absorber. Superior interfacial hole extraction and transfer capabilities were achieved using the SWCNT bridges, which outperformed the MWCNTs in charge transport and uniform network properties. Hence, the device showed remarkable stability and a power conversion efficiency of 15.73%. For all of their inorganic CsPbI_3_-based devices, researchers used PEI/CNTs, or polyethyleneimine-functionalized carbon nanotubes, as connections (Figure 8a–c). A reduction in the interface resistance and neutralization of surface trap states in the perovskite material was caused by the presence of many amines’ functional groups on the PEI/CNT bridge. The power conversion efficiency increased to 10.55% and the fill factor to 0.71 with the addition of PEI/CNTs, outperforming the device without PEI/CNTs, which achieved a PCE of 7.41% and an FF of 0.56. For PSC efficiencies and stabilities to be affected by the interfacial layers, their existence is critical. The interfaces of perovskites with hole transport layers, metal electrodes, and carbon have all benefited from the introduction of carbon nanotubes as intermediary layers. Another great way to increase the effectiveness of charge collection is to develop hybrid layers by integrating other substances into carbon nanotubes, such as polymers and two-dimensional graphene. Additional capabilities, such as adjusting perovskite’s morphology, reducing surface defects, and preventing moisture penetration, may be achieved by functionalized carbon nanotubes that contain the necessary groups. Devices benefit from these qualities, which increase their stability and efficiency [180]. A detailed comparison of the latest CNT-based PSCs is given in Table 3

### 5.5. CNT-Based Transparent Electrode

In order to guarantee that sunlight reaches light absorbers with minimal or no energy loss, solar cells require extremely transparent electrodes. A transparent electrode’s electrical conductivity and transmittance have a significant effect on how well solar cells operate in photovoltaic applications [181]. Due to their excellent optoelectronic qualities and well-established production methods, transparent and conductive doped metal oxides, like ITO and FTO, are the most widely used transparent electrodes in solar cells. However, there are still a number of disadvantages for their large-scale applications, including the scarcity and high cost of indium, their high production costs, and their brittle nature [182].

The use of MoO_3_-doped carbon nanotube top electrodes in perovskite solar cells leads to enhanced hole transport, p-doping, and energy-level alignment. The ideal thickness of the MoO_3_ layer is 8 nm in order to reduce the resistance of the carbon nanotube electrode sheet without causing any harm to the perovskite film. The addition of acid to carbon nanotube top electrodes results in a reduction in sheet resistance by one-third, surpassing their previous performance. MoO_3_ decreases the Fermi level of the carbon nanotube electrode, enhancing the alignment of energy levels and the effectiveness of hole transfer. When carbon nanotubes are coated with 2,2′,7,7′-tetrakis[N,N-di(4-methoxyphenyl)amino, MoO_3_ crystallizes, and hole collection is enhanced. The power conversion efficiency and transmittance at 1000 nm of semi-transparent perovskite solar cells including MoO_3_-doped carbon nanotube electrodes are reported to be 17.3% and 60%, respectively. Perovskite solar cells are often used in conjunction with silicon solar cells due to their infrared transparency. The efficiency of the technology, which stands at 23.7%, surpasses that of indium tin oxide tandem solar cells [183].

The study demonstrated the feasibility of constructing perovskite solar cells (PSCs) with high-performance, solution-processed, double-walled carbon nanotube (CNT) electrodes utilizing a parylene-C substrate that is both ecologically safe and biocompatible. A more robust p-doping effect, and consequently, increased conductivity of the CNTs were achieved by inducing vertical separation of the binders from the CNTs using a new inversion transfer technique. The resultant foldable devices demonstrated an exceptional mechanical stability, withstanding over 10,000 folding cycles at a radius of 0.5 mm, and a power conversion efficiency of 18.11 percent, the highest recorded among PSCs based on CNT transparent electrodes to date. The potential of the solution-processable nanocarbon electrode was assessed by fabricating solar modules using exclusively laser scribing techniques [184].

**Table 3:** CNT-based PSC materials for clean energy production with high efficacy.

Nanocomposite	Voc (V)	PCE (%)	Jsc (mA/cm^2^)	FF		Ref.
Glass/FTO/CH_3_NH_3_PbI_3_/Spiro-OMeTAD/Compact TiO_2_/CNT	0.88	6.87	15.46	0.51	HTL	[185]
FTO/TiO_2_/MAPbI_3_/MWCNTs/gold	1.100	18.3	19.192	0.86	HTL	[186]
PSC/CQDs/SWCNTs	0.49	14.7	17.59	0.87	HTL	[187]
Al/ZnO/TiO_2_/SWCNT/SnS/Pt	1.04	32.86	41.91	0.72	ETL/HTL	[21]
MWCNT/Cr_2_O_3_/PSCs	1.002	16.29	16.05	0.780	HTL	[188]
CNT-based perovskite solar cell	1.29	23.89	19.488	0.87	Semi-transparent CNT layer electrode	[189]
SWCNT/GaAs/vdW	0.64	11.24	24.70	0.71	ETL/HTL	[190]
NiO_x_/CNT/P_3_HT	0.99	11.00	19.40	0.57	HTM	[191]
SWCNTs/DEME-TFSI IL/MWCNT	0.8	13.2	14	0.40	ETL	[192]
CNT/IL	0.6	25.2	0.7	0.11	Electrical double layer	[193]
PSCs/CNT	0.98	16.17	15.7	0.68	Hole transportation material	[194]
PSCs/TiO_2_/CNT	1.046	17.4	22.50	0.72	ETL	[161]
Multi-walled nanotubes	0.85	52.8	10.86	0.42	HTL	[195]
NiO_x_/CNTs/PSCs	1.01	17.72	21.6	0.15	HTL	[132]
FTO/c-TiO_2_/m-TiO_2_/CH_3_NH_3_PbI_3_/CZTS-MWCNT/MWCNT)	0.95	6.8	10.95	0.58	HTM	[196]
CNT/PScs	1.01	14.1	19.9	0.69	HTM	[197]
FTO/SWCNT/TiO_2_	0.82	7.2	14.0	0.63	ETL	[198]
Spiro-OMeTAD + CNT:TiO_2_	1.11	24.24	21.53	0.75	HTL	[199]
CNTs-spiro	0.96	15.9	10.45	0.65	HTL	[200]
MWCNT:NiO in spiro-OMeTAD	1.17	24.91	22.73	0.78	Perovskite/HTL interface	[201]
f-NiO_x_ + CNT	0.97	18.20	11.36	0.64	HTL	[202]
TO/SnO_2_/Al_2_O_3_/CsFAMA:CNT:TiO_2_/(BA)_2_PbI_4_/spiro-OMeTAD/Au	1.20	24.16	0.76	22.7	Perovskite/HTL interface	[203]
PTAA-CNT	1.045	21.70	15.41	0.71	HTL	[204]
NiO_x_/CNTs	0.97	20.65	16.21	0.75	HTL	[132]
TFMS-doped DWNT/PTAA	1.02	21.4	17.2	0.77	Transparent electrode	[205]
FTO/c-TiO_2_/ms-TiO_2_•SWCNT/MAPbI_3_/PTAA/Au	1.10	23.60	20.4	0.79	ETL	[162]

## 6. Conclusions and Prospective

Because of its useful features and rapid efficiency gains, perovskite solar cells (PSCs) based on metal halides have attracted a lot of interest from the photovoltaic sector. Perovskite solar cell designs have found several uses for one-dimensional carbon nanotubes (CNTs) due to their exceptional electrical, optical, and mechanical properties. Among these parts are charge transporters, interface modifiers, perovskite additives, methods for transporting holes, and electrodes that collect charges. Including CNTs in these parts has a lot of promise for making PSCs that work well and stay put. Investigating the potential of carbon nanotubes in perovskite solar cells is an emerging field of research; despite notable advancements in this area in recent years, we still need to handle and investigate several critical concerns.

Carbon nanotubes are essential in photovoltaic solar cells, because they are both transparent and conductive, allowing sunlight to reach the perovskite light absorbers with little energy loss. This study mainly aimed to investigate SWCNTs (single-walled carbon nanotubes) and their characteristics. Nevertheless, the research shows that SWCNT sheets do not have enough transmittance and conductivity to be used in perovskite solar cell applications. Because they are more conductive than semiconducting or mixed nanotubes, high-purity metallic single-walled carbon nanotubes are highly sought after and may be obtained by synthesis or using post-treatment procedures. Further research is necessary to build high-performance PSCs, and the full potential of DWCNTs in PSCs has not been fully achieved yet. If we want to increase the conductivity and transmittance of CNTs, we need to find the right dopants that would have long-lasting effects. Before adding any more layers, one should think about how smooth the surface of the CNT transparent films is and whether or not researchers will work with the chosen charge connections at the interface. An efficient way to meet these objectives is to combine carbon nanotubes with other molecules, including conducting polymers. When it comes to creating efficient semitransparent devices and perovskite-based tandem solar cells, carbon nanotubes (CNTs) might be the ideal transparent top contacts. Improvements in integrated solar cells and photovoltaic technology’s power conversion efficiency are dependent on this line of inquiry.

In addition, perovskite solar cells’ enhanced electron transport layers (ETLs) using carbon nanotubes have shown a favorable performance. It would be very advantageous to have a deeper understanding of how the structural properties of carbon nanotubes impact device performance. One major problem with single-walled carbon nanotubes is that they are not compatible with other materials because of their unique electrical band structures, chemical compositions, and handedness. It is critical to have a comprehensive grasp of how these changes in properties affect the efficacy and longevity of PSCs. Carbon nanotubes added to existing HTLs or built into double HTLs may improve the charge carrier dynamics and device stability. It is critical to comprehend the role of carbon nanotubes in charge extraction and/or recombination when they are incorporated into organic or inorganic hole transport layers. The expansion of composite HTL systems benefits from this comprehension. An exciting new field of study is the double hole transport layer system, which combines various hole-transporting materials with polymer-wrapped, single-walled carbon nanotubes [206]. This strategy makes use of the special qualities of these one-of-a-kind combos. Nevertheless, further research is required to compare and clarify the precise impacts of encasing inert or active polymers on other kinds of SWCNTs, such as m-SWCNTs, s-SWCNTs, and mixtures of the two. Furthermore, CNT interlayers may prevent charge recombination and improve rapid charge extraction. Careful investigation of the charge transport materials’ and carbon nanotubes’ interactions with perovskites is essential. The charge dynamics process of perovskite solar cells might be enhanced by these interactions.

Additionally, perovskite film crystallization and form may be controlled by functionalized single-walled carbon nanotubes and multi-walled carbon nanotubes. A complete framework to aid in the rational choice and design of suitable functional groups for carbon nanotubes is currently lacking. Furthermore, there is a need for immediate and further research into the function of CNT additions, since our current knowledge is inadequate. Maintaining a steady stream of research into active functional groups and developing suitable methods for functionalizing CNTs while reducing their negative effects is essential. Further investigation is needed to understand how carbon nanotubes and the functional groups on their surfaces affect the growth and nucleation kinetics, as well as the trap states, of the crystallized perovskite films that are produced. In particular, improving the film quality and achieving stable, highly effective perovskite solar cells requires understanding the relationship between functionalized carbon nanotubes and device durability and how CNTs affect the basic principles of perovskites (such as optical, electrical, and charge transport properties). To shed light on these questions, improved experimental characterizations may make use of both in situ and ex situ methodologies, in addition to theoretical computations.

## Figures and Tables

**Table 1:** Advantages and disadvantages of different synthesis methods CNTs.

Methods	Merits	Demerits	References
Laser ablation	The incorporation of a metal catalyst into the carbon target leads to the synthesis of single-walled carbon nanotubes (SWCNTs) characterized by a narrow diameter distribution and elevation.	It is unsuitable for large-scale manufacturing.	[82]
Chemical vapor deposition	An extensive technique that also demonstrates how a multivariable process can be adjusted in a number of ways, including alcohol catalytic CVD, high-pressure CO disproportionation, plasma-enhanced CVD, thermochemical CVD, aerogel supported CVD, aerosol-assisted CVD, and hybrid laser-assisted thermal CVD.	Process parameter modifications are required to regulate the diameter distribution and yield of SWCNTs.	[82]
Electric arc discharge	Because of the high temperature employed, it is possible to make nanotubes with few structural errors. It is also an easy and affordable method that can yield a huge number of nanotubes.	The regulation of chirality in CNTs is not possible. If metal catalysts are used, it is necessary to purify the synthesized nanotubes. This procedure requires a high temperature.	[83]
	PECVD enables the formation of carbon nanotubes (CNTs) at much lower temperatures, sometimes even at room temperature, in comparison to thermal CVD (700–1000 °C). One potential avenue for exploration is the use of heat-sensitive substrates such as polymers or flexible electronics. The use of plasma in PECVD facilitates a heightened level of reactivity, hence enabling the improved manipulation of the properties shown by the carbon nanotubes (CNTs). The manipulation of plasma characteristics has the ability to affect the chirality, or handedness, of carbon nanotubes (CNTs), therefore affecting their electrical properties.	The use of high-energy plasma has the potential to induce the development of flaws inside carbon nanotubes (CNTs), which may have implications for their electrical and mechanical characteristics.	[84]
Catalyst method	It requires a low temperature.	It produces a low yield.	[57]
Arc discharge	It involves the mass production of carbon nanotubes (CNTs) and fullerenes.	It is a high-temperature process.	[85]
Electrocatalysis	This method has the following: a straightforward experimental setup; a controllable synthesis process through the use of electrolysis modes; inexpensive raw materials; controllable CNT structures; and the ability to dope carbon phases and morphologies in a single step through the optimization of electrolysis conditions and the electrolytic bath composition. Comparing electrolysis to other common CNT synthesis methods, this method is less expensive.	There are two primary issues with this approach, taking into account its drawbacks. The first isthe issue with the graphite cathode breaking during the electrolysis process, and the second is the buildup of electrolysis byproducts in the bath, including carbon nanomaterials (the cathode), alkaline metal (the anode), and chlorine gas (the anode). These two issues hinder the ongoing performance and lead to process instability.	[86]
Hydrothermal/solvothermal	When compared to previous techniques of CNT synthesis, this method has three major advantages. First of all, the starting materials are readily obtained and stable at room temperature; second, CNT development may be achieved using this method even at low temperatures of 150–180 °C; and third, a hydrocarbon gas or carrier gas is not needed for the CNT growth process.	It produces a low yield.	[86]
